# Soluble Triggering Receptor Expressed on Myeloid Cells 2 From Cerebrospinal Fluid in Sleep Disorders Related to Parkinson’s Disease

**DOI:** 10.3389/fnagi.2021.753210

**Published:** 2021-09-29

**Authors:** Mingshu Mo, Yuting Tang, Lijian Wei, Jiewen Qiu, Guoyou Peng, Yuwan Lin, Miaomiao Zhou, Wei Dai, Zhiling Zhang, Xiang Chen, Hanqun Liu, Liuyan Ding, Panghai Ye, Yijuan Wu, Xiaoqin Zhu, Zhuohua Wu, Wenyuan Guo, Pingyi Xu

**Affiliations:** ^1^Department of Neurology, The First Affiliated Hospital of Guangzhou Medical University, Guangzhou, China; ^2^Department of Physiology, School of Basic Medical Sciences, Guangzhou Medical University, Guangzhou, China

**Keywords:** Parkinson’s disease, TREM2 (triggering receptor expressed on myeloid cells), biomarker, sleep disorder (SD), cerebrospinal fluid (CSF)

## Abstract

**Background:** Triggering receptor expressed on myeloid cells 2 (TREM2) is a microglial receptor exclusively expressed in the central nervous system (CNS). It contributes to abnormal protein aggregation in neurodegenerative disorders, but its role in Parkinson’s disease (PD) is still unclear.

**Methods:** In this case-control study, we measured the concentration of the soluble fragment of TREM2 (sTREM2) in PD patients, evaluated their sleep conditions by the PD sleep scale (PDSS), and analyzed the relationship between sTREM2 and PD symptoms.

**Results:** We recruited 80 sporadic PD patients and 65 healthy controls without disease-related variants in TREM2. The concentration of sTREM2 in the CSF was significantly higher in PD patients than in healthy controls (*p* < 0.01). In the PD group, the concentration of sTREM2 had a positive correlation with α-syn in the CSF (Pearson *r* = 0.248, *p* = 0.027). Receiver operating characteristic curve (ROC) analyses showed that sTREM2 in the CSF had a significant diagnostic value for PD (AUC, 0.791; 95% CI, 0.711–0.871, *p* < 0.05). The subgroup analysis showed that PD patients with sleep disorders had a significantly higher concentration of sTREM2 in their CSF (*p* < 0.01). The concentration of sTREM2 in the CSF had a negative correlation with the PDSS score in PD patients (Pearson *r* = −0.555, *p* < 0.01). The ROC analyses showed that sTREM2 in the CSF had a significant diagnostic value for sleep disorders in PD (AUC, 0.733; 95% CI, 0.619–0.846, *p* < 0.05).

**Conclusion:** Our findings suggest that CSF sTREM2 may be a potential biomarker for PD and it could help predict sleep disorders in PD patients, but multicenter prospective studies with more participants are still needed to confirm its diagnostic value in future.

## Introduction

Parkinson’s disease (PD) is a common neurodegenerative disease that is characterized by an impaired dopaminergic (DA) neurotransmitter system, aggregation of α-synuclein (α-syn) and microglial neuroinflammation-mediated neuroinflammation in the midbrain (Block and Hong, 2005; Wang et al., 2015; Rai and Singh, 2020; Rai et al., 2021). In addition to motor symptoms, PD patients suffer from non-motor disorders such as sleep disorders (Stefani and Högl, 2020). Sleep disorders affect 60–98% of PD patients as one of the most common non-motor symptoms and it often appears as the first symptom (Chaudhuri et al., 2006; Stefani and Högl, 2020). The regulation of sleep depends on the comprehensive functions of multiple brain regions and a series of neurotransmitters, including dopamine, 5-hydroxytryptamine, norepinephrine, and other PD-related molecules (Alexandre et al., 2006; Meles et al., 2017; Oh et al., 2018; Hayat et al., 2020). The mechanism of sleep disorders in PD patients may involve the loss of DA neurons and the deposition of α-syn in the brain stem, basal ganglia and hypothalamus (Dolores et al., 2016; Doppler et al., 2017; Garrido et al., 2020; Stefani et al., 2021). It has been suggested that sleep disorders have a certain value for the prediction and diagnosis of PD (Barasa et al., 2021).

Microglia perform functions including monitoring changes in neuronal activity, regulating learning and memory, and acting as local phagocytes or damage sensors in the brain (Colonna and Butovsky, 2017). Microglia engulf extracellular aggregated α-syn mediated by special receptors such as lymphocyte activation gene 3 (Lag-3) (Guo Y. et al., 2019; Gu et al., 2021). The dysfunction of microglia impairs the clearance of extracellular α-syn, which may lead to neurodegeneration of DA neurons in PD (Filippini et al., 2019; Li et al., 2019). Triggering receptor expressed by myeloid cells 2 (TREM2) is expressed exclusively by microglia in the central nervous system (CNS) (Dempsey, 2015). Microglia may recognize abnormal extracellular signals through the TREM2 receptor, a process that participates in neuron degeneration (Udeochu et al., 2018; Lee et al., 2021). In PD animal models, TREM2 deficiency can aggravate α-syn-induced neurodegeneration and neuroinflammation in the CNS, and its overexpression remarkably reduces toxicity-induced apoptosis of DA neurons (Ren et al., 2018; Guo Y. et al., 2019). This result suggests that TREM2 may enhance microglial phagocytosis function and inhibit neuroinflammation and neurodegeneration in the brain (Leyns et al., 2017; Udeochu et al., 2018; Guo Y. et al., 2019). In clinical studies, single nucleotide polymorphisms (SNPs) of exon 2 of the TREM2 gene, including rs75932628, rs143332484 and rs2234253, which are closely related to the genetic susceptibility to Alzheimer’s disease (AD) (Sims et al., 2017; Zhou et al., 2019), have not been demonstrated to show a relationship with PD (Li et al., 2016; Mengel et al., 2016). The extracellular segment of TREM2, recoded by exon 2, can be proteolytically cleaved and released into the extracellular space as soluble fragments (sTREM2) in the cerebrospinal fluid (CSF) (Kleinberger et al., 2014; Piccio et al., 2016). sTREM2 is thought to be an ideal biomarker for microglia-involved neurological disorders (Bekris et al., 2018).

**FIGURE 1:**
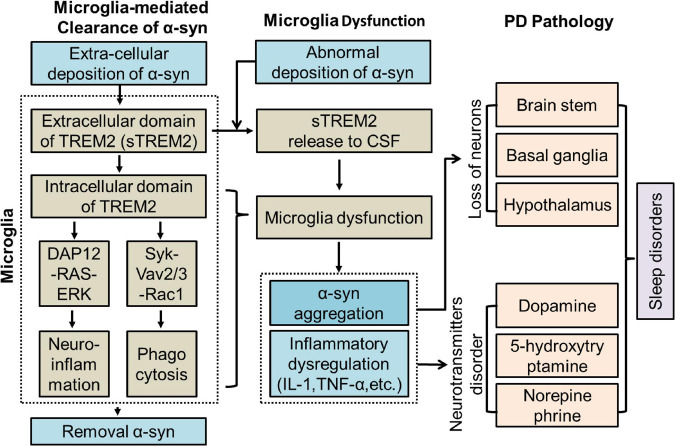
Potential role of TREM2 in PD. CSF, Cerebrospinal fluid; DAP12, DNAX-activating protein of 12 kDa; ERK, extracellular signal-regulated kinase 3; IL-1, interleukin-1; PD, Parkinson’s Disease; sTREM2, soluble TREM2; TNF-α, tumor necrosis factor-α.

Recent studies have shown that the concentration of sTREM2 in the CSF is increased in individuals with neuroinflammation in the CNS (Bekris et al., 2018; Ren et al., 2018). The concentration of sTREM2 in the CSF is closely correlated with aging (Henjum et al., 2016; Mecca et al., 2018). A higher concentration of sTREM2 was found to be associated with poor sleep characteristics in amyloid-positive individuals (Boespflug and Iliff, 2018; Wilckens et al., 2018; Belsare et al., 2020; Hu et al., 2021). sTREM2 in the CSF has a close relationship with tau-mediated neurodegenerative diseases such as AD (Piccio et al., 2016; Henjum et al., 2018; Sayed et al., 2018). The concentration of sTREM2 in the CSF was found to be elevated in PD patients with a positive-tau biomarker signature (Wilson et al., 2020). The α-syn protein shares similar pathobiology to tau and amyloid and is prone to pathological misfolding and aggregation in the CNS (Veys et al., 2021). The relationship between sTREM2 and α-syn is still not clear. Thus, in this study, we measured the concentrations of sTREM2 in the CSF, evaluated its correlation with PD and sleep disorders, and speculated its role in PD (Figure 1).

## Materials and Methods

### Subjects

A total of 145 Han Chinese subjects, including 80 sporadic PD patients and 65 healthy controls matched by age, sex, and ethnicity, were recruited from the First Affiliated Hospital of Guangzhou Medical University and Guangdong 999 Brain Hospital from January 2017 to August 2021. PD was diagnosed according to the United Kingdom Parkinson’s Disease Society Brain Bank clinical diagnostic criteria for Parkinson’s disease. According to the results of the PD sleep scale (PDSS) (Tse et al., 2005), the PD patients were divided into a PDSS ≥ 90 group with no or mild sleep disorder and a PDSS < 90 group with moderate or severe sleep disorder. The global cognitive functions of all participants were assessed by the Montreal Cognitive Assessment test (MoCA) (Nasreddine et al., 2005). The conditions of the PD patients were evaluated by the International Parkinson and Movement Disorder Society (MDS)-sponsored revision of the Unified Parkinson’s Disease Rating Scale (UPRDS) and the Hoehn and Yahr stage (H&Y). Healthy control participants without a history of PD, other neurodegenerative diseases or chronic neuropsychiatric disorders were recruited from the surrounding community. The study protocols were approved by our institutional review boards. All samples were analyzed after obtaining informed consent from the participants.

### Plasma and Cerebrospinal Fluid Collection

Peripheral blood samples were collected from all participants within 2 weeks after CSF collection, and genomic DNA was extracted by a Blood DNA Kit (QIAGEN, Dutch). Exon 2 of TREM2 was amplified by polymerase chain reaction (PCR) in an ABI 7900HT Fast Real-Time PCR system (Applied Biosystems, United States). The primers were designed by Primer Software Version 5.0 (Premier, Canada) as follows: 5′-3′ AAGTGCCTCCAGAATAGACC, 3′-5′ CCTTGGTAGCCCTGAGTAG. Sanger sequencing was used to investigate polymorphisms in exon 2 of the TREM2 gene using an ABI 3730XL system at Beijing Genomics Institute. Variant frequencies of TREM2 were compared by Fisher’s exact test. The CSF samples without hemolysis or discoloration were collected in polypropylene tubes, stored at −80°C, and subjected to a maximum of two freeze-thaw cycles prior to the detection of sTREM2 or α-syn.

### Cerebrospinal Fluid Soluble Triggering Receptor Expressed on Myeloid Cells 2 and α-Syn Measurement

The concentrations of sTREM2 and α-syn were detected by enzyme-linked immunosorbent assay (ELISA) as previously described (Peng et al., 2020). After the freeze-thaw cycles, the Human TREM2 DuoSet ELISA (R&D Systems: #DY1828–05, Minneapolis, MN, United States) and Human alpha-Synuclein DuoSet ELISA (R&D Systems: #DY1338–05, Minneapolis, MN, United States) were used to measure the concentration of sTREM2 and α-syn in the CSF according to the manufacturer’s instructions (Peng et al., 2020). Briefly, 96-well streptavidin plates were blocked with 3% bovine serum albumin (BSA) and coated with the capture antibodies. The capture antibodies were anti-human TREM2 (#844598, R&D) or anti-α-syn (#844224, R&D). The samples and recombinant human TREM2 (#844600, R&D) or the α-syn (#844226, R&D) standard were incubated overnight with the capture antibodies. After three washes with phosphate-buffered saline (PBS)/0.05% Tween-20 (PBS-T) buffer, a biotinylated mouse anti-human TREM2 (#844599, R&D) or anti-α-syn (#844225, R&D) mAb was added and incubated for 2 h. Then, the detection was performed with a SpectraMax M2 plate reader (Molecular Devices, United States). Recombinant soluble human TREM2 or α-syn was used to construct the ELISA standard curve. The sample was discarded if its intraplate coefficient of variation (CV) > 15% or intraplate CV > 5%. Samples were distributed in a randomized manner and detected by an operator blinded to the clinical information. Samples were re-measured with the same reagents on the same day.

### Statistical Analyses

Differences in the demographic data between the PD and control groups were assessed using the Chi-square test for categorical variables and the *t*-test for the age variable. The α-syn and sTREM2 concentration data had skewed distributions and were analyzed by Mann-Whitney *U* tests. Differences in SNPs of TREM2 with rare frequencies were analyzed using Fisher’s exact test. Receiver operating characteristic (ROC) curve analysis was used to evaluate the diagnostic performance of the CSF sTREM2 concentration by the area under the ROC curve (AUC). The Pearson correlation coefficients were used for correlational analyses. The *p*-values were two-tailed since all hypotheses tested were two-sided, and a significance level of α = 0.01 was selected. GraphPad Prism (GraphPad Software Inc., Version 6, United States) and SPSS version 21.0 (SPSS Inc., United States) were used for the analyses.

## Results

### Characteristics of the Participants

The demographic characterization of the PD cases and controls is presented in Table 1. A total of 80 sporadic PD patients and 65 age- and sex-matched controls were recruited as two groups. The PD group recruited patients diagnosed at an early H&Y stage of disease (Unpaired *t*-test, 1.98 ± 0.43 years) with a short disease duration (3.1 ± 1.5 years) and a moderate MDS-UPDRS-III score (30.39 ± 15.25). Compared with the controls, the PD group had no significant difference in MoCA scores (Unpaired *t*-test, *p* = 0.032), which is used to evaluate global cognition and executive function. As expected, performance on the PDSS, a test sensitive to assessing sleep disorders in subjects with PD, was significantly lower in the PD group than in the healthy control group (*p* < 0.001). The PD groups had a significantly lower concentration of α-syn in the CSF (Mann–Whitney test, *p* < 0.001) and a higher concentration of sTREM2 in the CSF (Mann–Whitney test, *p* < 0.001). Exon 2 encodes the main body of the extracellular segment in TREM2 that can be proteolytically cleaved and released into CSF as a soluble peptide named sTREM2. To avoid the side effects of gene mutations, targeted sequencing of exon 2 of TREM2 was performed (Piccio et al., 2016). Three variants (p. S81N, p. R47H, p. G58D) were shared by the control and PD groups, while the other two variants (p. A105 V, p. A62H) were only detected in PD patients. The percentage of variants on exon 2 of TREM2 was not significantly different between the two groups (Fisher’s test, *p* = 0.073).

**TABLE 1:** The demographic characterization of PD cases and controls.

**Demographics**	**Control**	**PD**	***p***
Number	65	80	**−**
Age, years, mean (SD)	62.49 (6.90)	63.59 (8.50)	0.407^a^
Gender, female/male	26/39 (0.67)	32/48 (0.67)	1.000^b^
Disease duration, years, mean (SD)	−	3.16 (1.52)	−
MoCA score, mean (SD)	26.32 (1.95)	25.73 (1.88)	0.032^a^
H&Y stage, mean (SD)	−	1.98 (0.43)	−
MDS-UPDRS-III, mean (SD)	−	30.39 (15.25)	−
PDSS score [median (range)]	133 (58–150)	90.50 (35–147)	<0.001^c^**
CSF α-synuclein, pg/mL [median (range)]	1979.43 (496.1–3457.7)	1702.96 (608.72–4556.76)	<0.001^c^**
CSF sTREM2, pg/mL [median (range)]	287.24 (92.65–515.57)	403.43 (79.41–959.23)	<0.001^c^**
Variants in exon2 of TREM2	3	5	0.073^d^

*^a^Unpaired *t*-test.*

*^b^Chi-square test.*

*^c^Mann–Whitney test.*

*^d^Fisher’s precision probability test.*

**P < 0.01 versus control, ns = not significant.

*CSF, Cerebrospinal fluid; PD, Parkinson’s Disease; SD, standard deviation; H&Y stage, Hoehn-Yahr stage; sTREM2, soluble TREM2; PDSS, PD sleep scale.*

### Cerebrospinal Fluid Soluble Triggering Receptor Expressed on Myeloid Cells 2 Has Potential Value as a Biomarker of Parkinson’s Disease

**FIGURE 2:**
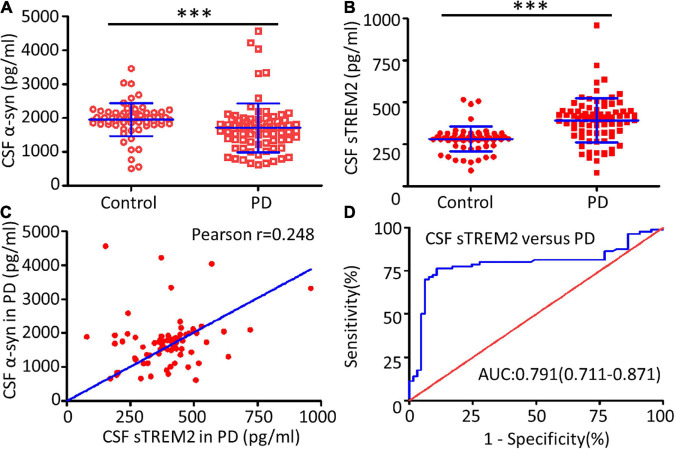
Correlation between CSF sTREM2 and PD. The concentrations of α-syn **(A)** and sTREM2 **(B)** in the CSF from PD patients and controls were determined by ELISA. The samples consisted of 80 PD patients and 65 controls. The sTREM2 values are shown as medians ± interquartile ranges with blue bars. The Mann–Whitney test was used for comparisons. **(C)** A significant association was observed between sTREM2 and α-syn in the CSF from PD patients (Pearson *r* = 0.248, *p* = 0.027) by Pearson correlation coefficient analysis. **(D)** ROC curve analysis of CSF sTREM2 in discriminating PD patients from healthy controls. The area under the curve was 0.791 (95% CI = 0.711–0.871). ^∗∗∗^*p* < 0.001 versus control.

The TREM2 receptor may regulate the microglial response to engulf aggregated α-syn and it could be used as a sensor in PD. CSF sTREM2, reflecting microglial function in the CNS, was evaluated as a characteristic hallmark of AD (Wood, 2017). Compared with the healthy control group, the PD group had a significantly decreased concentration of α-syn (Mann–Whitney *U* test, *p* < 0.001) and an increased concentration of sTREM2 (Mann–Whitney *U* test, *p* < 0.001) in the CSF (Figures 2A,B). The increasing trend of CSF sTREM2 was positively correlated with the trend of CSF α-syn in PD patients (Pearson *r* = 0.248, *p* = 0.027, Figure 2C). CSF α-syn has been proven to be a useful biomarker for the diagnosis of PD, suggesting that CSF sTREM2, closely related to CSF α-syn, may also have a similar function as a biomarker. The ROC curve analysis revealed that CSF sTREM2 could differentiate PD patients from age-matched healthy controls, with an area under the curve (AUC) of 0.791 (95% CI = 0.711–0.871, *p* < 0.001, Figure 2D).

### Cerebrospinal Fluid Soluble Triggering Receptor Expressed on Myeloid Cells 2 Concentration Is Positively Associated With Sleep Disorders in Parkinson’s Disease

Sleep disorders, as one of the earliest non-motor symptoms of PD, may show an increased prevalence or a worsening of symptoms with disease progression (Stefani and Högl, 2020). According to the PDSS score used for the assessment of sleep disorders, the PD patients were further subdivided into two subgroups: the PDSS ≥ 90 group with no or mild sleep disorders and the PDSS < 90 group with moderate or severe sleep disorders. The two groups differed in disease duration (Mann–Whitney *U* test, *p* < 0.001), with a higher concentration of CSF sTREM2 in the PDSS ≥ 90 group (Mann–Whitney test, *p* < 0.001), but there was no difference in age (Unpaired *t*-test, *p* = 0.016), sex (Chi-square test, *p* = 0.491), H&Y stage (Mann–Whitney *U* test, *p* = 0.852) or MDS-UPDRS III score (Mann–Whitney *U* test, *p* = 0.410, Table 2).

**TABLE 2:** The demographic characterization of PD with a sleep disorder.

**Demographics**	**PD with PDSS < 90**	**PD with PDSS ≥ 90**	***P* value**
Number	40	40	−
Age, years, mean (SD)	66 (8.50)	61 (7.86)	0.016^a^
Gender, female/male	18/22 (0.82)	14/26 (0.54)	0.519^b^
Disease duration, years, [median (range)]	4.00 (0.25–6.00)	2.80 (0.50–5.00)	<0.001^c^**
H&Y stage, [median (range)]	2.00 (1–2.5)	2.00 (1–2.5)	0.852^c^
MDS-UPDRS III, [median (range)]	24 (10–68)	25 (9–98)	0.410^c^
PDSS score, mean (SD)	67.23 (13.32)	109.35 (12.40)	<0.001^a^**
CSF sTREM2, pg/ml [median (range)]	443.25 (151.73–959.21)	371.55 (79.41–489.72)	<0.001^c^**

*^a^Unpaired *t*-test.*

*^b^Chi-square test.*

*^c^Mann–Whitney *U* test.*

**P < 0.01 versus control, ns = not significant.

*CSF, Cerebrospinal fluid; PD, Parkinson’s Disease; SD, standard deviation; H&Y stage, Hoehn-Yahr stage; sTREM2, soluble TREM2; PDSS, PD sleep scale.*

**FIGURE 3:**
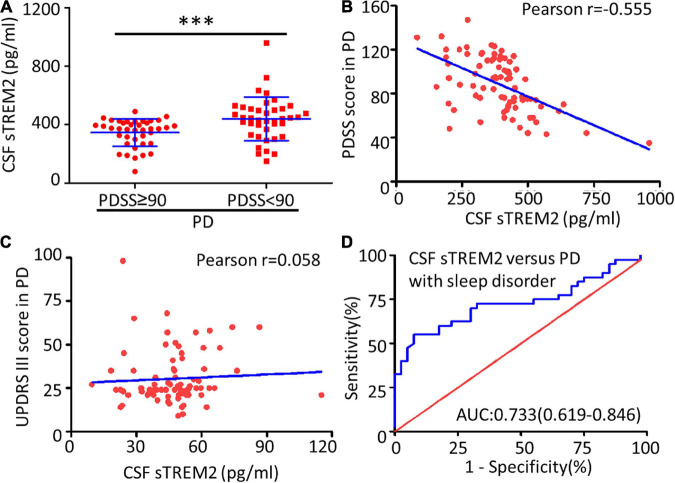
Correlation between CSF sTREM2 and PD with a sleep disorder. **(A)** The concentration of sTREM2 in CSF from PD patients with PDSS ≥ 90 (*n* = 40) or PDSS < 90 (*n* = 40) was determined by ELISA. The sTREM2 values are shown as medians ± interquartile ranges with blue bars. The Mann–Whitney test was used for comparisons. Pearson correlation coefficients were used. The concentration of sTREM2 in the CSF had a significant association with the PDSS score in PD patients (Pearson *r* = -0.555, *p* < 0.001) in **(B)** but not with the UPDRS III score (Pearson *r* = 0.058, *p* = 0.607) in **(C)**. **(D)** ROC curve analysis of CSF sTREM2 in discriminating moderate- or severe-sleep disorder (PDSS < 90) from no- or mild-sleep disorder with PDSS ≥ 90 in PD. The area under the curve was 0.733 (95% CI = 0.619–0.846). ^∗∗∗^*p* < 0.001 versus control.

Compared with the no or mild sleep disorder (PDSS ≥ 90) group, the PD patients with a moderate or severe sleep disorder (PDSS < 90) had a significantly increased concentration of sTREM2 in their CSF (Mann–Whitney test, *p* < 0.001, Figure 3A). The increasing trend of the CSF sTREM2 was negatively correlated with a closely decreasing trend of the PDSS score in PD patients (Pearson correlation analysis, Pearson *r* = -0.555, *p* < 0.001) but had no significant relationship with the UPDRS III score, which was used for the assessment of motor symptoms in PD (Pearson correlation analysis, Pearson *r* = 0.058, *p* = 0.607, Figures 3B,C). The ROC curve analysis revealed that CSF sTREM2 could differentiate PD patients with a moderate or severe sleep disorder from those with no or mild sleep disorder, with an AUC of 0.733 (95% CI = 0.619–0.846, *p* < 0.001, Figure 3D). This result suggested that CSF sTREM2 may have potential as a biomarker for disease progression in PD.

## Discussion

The clinical presentation and progression of neurodegenerative disease shows substantial biological heterogeneity (Chen-Plotkin et al., 2018). Biological function-based biomarkers may help to differentiate subgroups of PD by their rate of progression accompanied by motor or non-motor symptoms (Chen-Plotkin et al., 2018). In the present study, we measured the levels of sTREM2 in CSF, evaluated its association with PD onset, and found that CSF sTREM2 is a promising candidate biomarker for PD, with higher sTREM2 levels corresponding to lower α-syn levels in CSF at disease onset. In the subgroup analysis, we found that higher sTREM2 levels were correlated with worse sleep disorders, as measured by the PDSS scale. Taken together, our data suggest that sTREM2 levels in CSF may reflect the progression of PD, indicating it is a valuable biomarker.

TREM2 receptors are exclusively expressed on microglia in the CNS (Dempsey, 2015). sTREM2 in the CSF is a released extracellular segment of the single-pass transmembrane protein of TREM2 after proteolytic cleavage (Kleinberger et al., 2014; Piccio et al., 2016). The physiological- and disease-associated functions of sTREM2 are not clear. sTREM2 has been suggested to have protective functions on microglial activity and viability (Zhong et al., 2017). Before release from the membrane, sTREM2 acts as a receptor and recognizes and binds to special ligands, such as apoptotic neurons, misfolded proteins, and cellular debris (Yeh et al., 2017). After release into the CSF, sTREM2 can directly bind monomeric Aβ42 (mAβ42) and potently inhibit mAβ42 polymerization (Henjum et al., 2018). The concentration of sTREM2 in the CSF is a sensitive marker of microglial activation and was reported to correlate with the prevalence of tau-mediated AD (Henjum et al., 2018; Wilson et al., 2020). In addition, the CSF sTREM2 level reflects TREM2 expression, and TREM2-triggered microglial activity in the CNS is an ideal biomarker for microglia-involved disease (Brendel et al., 2017; Hu et al., 2021). High sTREM2 concentrations are found mainly in the preclinical stage of AD, frontotemporal dementia, vascular dementia, and aging adults (Henjum et al., 2016; Suárez-Calvet et al., 2016; Wang et al., 2020; van der Ende et al., 2021). α-syn in PD has similar mechanisms of aggregation and degradation as Aβ in AD (Rai and Singh, 2020; Rai et al., 2020; Cong et al., 2021; Rai et al., 2021). They share a GAV motif (VGGAVVAGV) in peptides which may be involved with initiating protein aggregation or fibrillization, and similar degradation mechanisms including intra- and extra- cellular aggregation, microglial activation and endocytosis (Lin et al., 2010; Schechter et al., 2020; Lashuel, 2021). It suggests that TREM2 may contribute to microglial recognition and endocytosis of α-syn. In our study, we found that the levels of sTREM2 in the CSF of PD patients were higher than those of controls and positively correlated with the levels of α-syn in the CSF, an association previously observed in PD patients (Peng et al., 2020). We found that an elevated concentration of sTREM2 in CSF may help to differentiate PD patients from healthy controls with an AUC of 0.791 (CI = 0.711–0.871). To avoid the effects of gene mutation, we sequenced exon 2 of the TREM2 gene encoding the sTREM2 peptides and found that the distribution of SNPs was not significantly different between the PD and control groups. Our study suggested that sTREM2 may be an ideal biomarker for PD diagnosis, but more supporting data from multicenter studies are still needed.

Sleep disorders include insomnia, daytime somnolence, rapid eye movement (REM) sleep behavior disorder, periodic limb movement, etc. (Tse et al., 2005). Sleep disorder, present in 64.1% of PD patients, was listed as the second most frequent non-motor symptom (Pont-Sunyer et al., 2015; Lysen et al., 2019). Shorter sleep duration and worse sleep quality are associated with a higher risk of parkinsonism in the first 2 years (Lysen et al., 2019). Sleep disorders appear at any stage of PD and become aggravated with disease progression (Videnovic, 2018). The pathogenesis of sleep disorders in PD is still not clear. The role of microglia in sleep disorders should not be underestimated. Microglial inflammatory responses, which are involved in neurodegenerative diseases such as PD and AD, can induce sleep disorders and are regulated by the circadian clock (Fonken et al., 2015). Peripheral blood samples from narcolepsy patients show increased levels of interleukin (IL)-6, tumor necrosis factor (TNF)-α, and major histocompatibility complex (MHC) class II, and reduced levels of chemokine receptor (CCR) 1 and CCR3, which are derived from microglia and macrophages, and IL-1 and TNF-α, which are well known to promote sleep in humans (Agnes et al., 2017). Another key factor related to sleep disorders is α-syn (Dolores et al., 2016). Abnormal α-syn, normally localized to presynaptic terminals of neurons, forms pathological amyloid aggregates deposited in multiple brain regions and induces abnormal neurotransmitter release, such as sleep-related interleukins (Dolores et al., 2016; Doppler et al., 2017; Garrido et al., 2020; Stefani et al., 2021). Phosphorylated α-syn deposits in the colon, saliva, glands, and skin can be used to differentiate subjects with isolated rapid eye movement sleep behavior disorder (RBD) from controls with 24–89% sensitivity and 78–100% specificity (Doppler et al., 2017). The TREM2 receptor is involved in the regulation of microglial function and α-syn cleaning (Guo Y. et al., 2019), but its relationship with sleep disorders in PD is not clear. In our study, we found that the levels of sTREM2 in the CSF among patients with worse sleep disorders were lower in PD and negatively correlated with the PDSS score, as was its association with α-syn in CSF. The elevated concentration of sTREM2 in the CSF can be used to differentiate PD patients with sleep disorders with an AUC of 0.733 (CI = 0.619–0.846). These results further support the relationship between sTREM2 and PD and suggest that sTREM2 may be useful to evaluate sleep disorders in PD progression.

## Conclusion

Taken together, we demonstrated that the concentration of sTREM2 in the CSF may represent a biomarker for monitoring the progression of PD, and an increased concentration of sTREM2 in the CSF was positively correlated with α-syn levels and sleep disorders. These findings may provide novel insights into a pivotal role for sTREM2, which serves as a biomarker of TREM2-mediated microglial function and can help to evaluate the development of PD.

## Data Availability Statement

The raw data supporting the conclusions of this article will be made available by the authors, without undue reservation.

## Ethics Statement

The studies involving human participants were reviewed and approved by Medical Ethics Committee of Guangzhou Medical University. The patients/participants provided their written informed consent to participate in this study.

## Author Contributions

MM and PX conceived and designed the study and interpreted the experiments. YT, LW, and WG performed the study and prepared the initial draft of the manuscript. MM, PX, XZ, ZW, YL, and YT supervised the project. WD, YW, JQ, GP, YL, MZ, WG, ZZ, LD, HL, PY, and XC provided the clinical samples and data. All authors read and approved the final submission.

## Conflict of Interest

The authors declare that the research was conducted in the absence of any commercial or financial relationships that could be construed as a potential conflict of interest.

## Publisher’s Note

All claims expressed in this article are solely those of the authors and do not necessarily represent those of their affiliated organizations, or those of the publisher, the editors and the reviewers. Any product that may be evaluated in this article, or claim that may be made by its manufacturer, is not guaranteed or endorsed by the publisher.
